# Systemic brain derived neurotrophic factor but not intestinal barrier integrity is associated with cognitive decline and incident Alzheimer’s disease

**DOI:** 10.1371/journal.pone.0240342

**Published:** 2021-03-04

**Authors:** Robin M. Voigt, Shohreh Raeisi, Jingyun Yang, Sue Leurgans, Christopher B. Forsyth, Aron S. Buchman, David A. Bennett, Ali Keshavarzian

**Affiliations:** 1 Division of Digestive Diseases and Nutrition, Department of Internal Medicine, Rush University Medical Center, Chicago, Illinois, United States of America; 2 Rush Alzheimer’s Disease Center, Rush University Medical Center, Chicago, Illinois, United States of America; 3 Department of Neurological Sciences, Rush University Medical Center, Chicago, Illinois, United States of America; Nathan S Kline Institute, UNITED STATES

## Abstract

The inflammatory hypothesis posits that sustained neuroinflammation is sufficient to induce neurodegeneration and the development of Alzheimer’s disease (**AD**) and Alzheimer’s dementia. One potential source of inflammation is the intestine which harbors pro-inflammatory microorganisms capable of promoting neuroinflammation. Systemic inflammation is robustly associated with neuroinflammation as well as low levels of brain derived neurotrophic factor (**BDNF**) in the systemic circulation and brain. Thus, in this pilot study, we tested the hypothesis that intestinal barrier dysfunction precedes risk of death, incident AD dementia and MCI, cognitive impairment and neuropathology. Serum BDNF was associated with changes in global cognition, working memory, and perceptual speed but not risk of death, incident AD dementia, incident MCI, or neuropathology. Neither of the markers of intestinal barrier integrity examined, including lipopolysaccharide binding protein (**LBP**) nor intestinal fatty acid binding protein (**IFABP**), were associated with risk of death, incident AD dementia, incident mild cognitive impairment (**MCI**), change in cognition (global or domains), or neuropathology. Taken together, the data in this pilot study suggest that intestinal barrier dysfunction does not precede diagnosis of AD or MCI, changes in cognition, or brain pathology. However, since MCI and AD are related to global cognition, the findings with BDNF and the contiguous cognitive measures suggest low power with the trichotomous cognitive status measures. Future studies with larger sample sizes are necessary to further investigate the results from this pilot study.

## Introduction

Many factors, including both genetic and environmental, contribute to the development of clinical and pathological phenotypes of Alzheimer’s disease (**AD**). The development of AD is likely the consequence of a myriad of factors such as disease, infection, stress, and environmental exposure to toxins. One concept that is gaining interest is the inflammatory hypothesis whereby sustained inflammation (e.g., IL-1β, IL-6, TNF-α, CRP) results in accumulation of amyloid-β and/or microglial activation sufficient to induce neurodegeneration [1]. Although studies report varying levels of neuroprotection with anti-inflammatory drugs (some show no protection) and interventions that reduce inflammation (e.g., horticultural therapy, mindfulness), evidence suggests that reducing inflammation may protect against or delay AD dementia [2–10]. Therefore, it is possible that conditions that promote systemic inflammation may promote the development and progression of AD.

One potential source of inflammation is the intestine which harbors a diverse collection of pro-inflammatory microorganisms including bacteria, viruses, and fungi. The intestinal barrier allows the absorption of water, electrolytes, and nutrients while at the same time preventing the pro-inflammatory contents of the intestine (e.g., lipopolysaccharide (**LPS**)) from reaching the systemic circulation where they can promote inflammation and the production of pro-inflammatory cytokines. Intestinal barrier dysfunction induces inflammation and promotes inflammation-mediated diseases [11–14]. Since LPS is found in the brain tissue of AD patients [15], it is possible that intestinal barrier dysfunction is a trigger for neuroinflammation and precedes cognitive dysfunction.

There is reciprocal relationship between inflammation and levels of brain derived neurotrophic factor (**BDNF**) whereby high levels of cytokines are reported to be associated with low BDNF [16]. BDNF is widely distributed throughout the central and peripheral nervous systems and is critical for the survival and function of neurons as well as memory formation and recall [17–24]. Studies show that BDNF is reduced in people with dementia, mild cognitive impairment (**MCI**), and AD and that higher BDNF in the brain [25–30] and systemic circulation [31–38] is associated with a slower rate of cognitive decline in individuals diagnosed with MCI or AD. A few studies have demonstrated that serum BDNF levels are associated with incident dementia [39], but there is a dearth of literature examining the co-morbid associations between intestinal barrier integrity and serum BDNF and if they are associated with AD dementia in older adults. To address these knowledge gaps, we integrated clinical data and postmortem brain histopathology indices with serum markers of barrier integrity and BDNF in older adults participating in the Religious Order Study (**ROS**). Specifically, this pilot study examined if systemic markers of intestinal barrier integrity (LPS binding protein (**LBP**), intestinal fatty acid binding protein (**IFABP**)) or serum BDNF levels collected from older adults without clinical evidence of AD dementia were associated with risk of death, incident AD dementia or MCI, or cognitive impairment. In decedents who had undergone brain autopsy, tissue was examined to determine if the markers of barrier integrity and BDNF were related to postmortem indices of AD-Related Dementia (**ADRD**) neuropathologies.

## Materials and methods

### Subjects

Data came from older adults participating in ROS, a community-based cohort study of chronic conditions of aging in older Catholic nuns, priests, and lay brothers recruited from approximately 40 groups across the United States [40, 41]. Participants agreed to annual clinical evaluations and blood draws, as well as brain donation at the time of death. Age in years, sex, and education were collected from self-reported information interview. This study was reviewed and approved by the Institutional Review Board of Rush University Medical Center. All participants signed an informed consent.

Serum samples used for the analyses were collected from all subjects with a clinical cognitive diagnosis of no cognitive impairment to determine if systemic biomarkers (IFABP, LBP, BDNF) were associated with the subsequent development of MCI or AD dementia. Subject characteristics are detailed in Table 1.

**Table 1:** Characteristics of study participants (n = 88).

	Mean (SD) or n (%)
Age, years^#^	76.398 (5.728)
Sex, male, n (%)	26 (29.55%)
Education, years	18.727 (3.473)
Length of follow up, years	13.692 (5.974)
Global cognition^#^	0.353 (0.453)
Episodic memory^#^	0.466 (0.479)
Perceptual orientation^#^	0.205 (0.694)
Perceptual speed^#^	0.386 (0.719)
Semantic memory^#^	0.288 (0.606)
Working memory^#^	0.299 (0.757)
Incident MCI, n (%)	65 (73.86%)
Incident AD, n (%)	41 (46.59%)
Death, n (%)	70 (79.55%)
Global AD pathology	0.719 (0.614)
β-amyloid	4.354 (4.250)
Tau tangles	8.081 (9.584)
Lewy bodies, n (%)^¶^	18 (20.45%)
BDNF*^#^	19,250.918 (5369.517)
IFABP*^#^	1,149.265 (1042.795)
LBP*^#^	24,746.246 (13287.815)

AD, Alzheimer’s disease; BDNF, brain derived neurotrophic factor; IFABP, intestinal fatty acid binding protein; LBP, lipopolysaccharide binding protein; MCI, mild cognitive impairment; SD, standard deviation.

^#^baseline data,

*raw data,

^¶^sample size = 67.

### Cognitive assessment and clinical diagnoses

Each year, cognitive tests were administered to subjects which were used to generate a composite measure of global cognition [40, 41]. The global cognition score was calculated as were the scores for five subdomains: episodic memory, semantic memory, working memory, perceptual speed, and perceptual orientation (visuospatial ability) scaled and centered by subtracting the baseline mean and dividing by the baseline standard deviation (**SD**). Clinical diagnosis was made each year. Dementia was determined using guidelines of the joint working group of the National Institute of Neurological and Communicative Disorders and Stroke and Alzheimer’s Disease and Related Disorders Association [42]. Individuals with cognitive impairment but without dementia were diagnosed as mild cognitive impairment (**MCI**), and the remainder were designated as no cognitive impairment (**NCI**) [43, 44]. Individuals with other dementias were not included in the study. After death, a neurologist blinded to autopsy data reviewed available cognitive and clinical data to assign a summary clinical diagnosis. The last valid diagnosis (NCI, MCI, or AD) following cognitive testing and clinical assessment was used to characterize each subject.

### Postmortem brain analyses

To date, 67 subjects had brain autopsies with neuropathologic assessments. Brain removal, tissue sectioning and preservation, and a uniform examination with quantification of postmortem outcomes followed previously published protocols [40, 41]. Postmortem measures included presence of pathologic AD based on National Institute on Aging criteria, a continuous measure summarizing the burden of AD pathology, as well as β-amyloid load and tangles. In addition, the presence of Lewy body disease (**LBD**) pathology was recorded.

### Serum IFABP, LBP, and BDNF

Serum samples were evaluated for the following biomarkers. *(1) IFABP*. IFABP is an intracellular protein specifically and abundantly expressed in epithelial cells in the intestine. When intestinal mucosa damage occurs, IFABP is released into the circulation [45], whereby higher levels of systemic IFABP indicate intestinal barrier damage [46]. IFABP levels in the serum were measured via ELISA per manufacturer instructions (Hycult Biotech Inc, Plymouth Meeting, PA, USA). *(2) LBP*. LBP plays a role in the innate immune response by binding to LPS, a lipid present in the outer membrane of all Gram-negative bacteria, to facilitate the association between LPS with CD14 resulting in the release of cytokines in response to LPS [47]. LBP levels in the serum were measured via ELISA per manufacturer instructions (Hycult Biotech Inc, Plymouth Meeting, PA, USA). *(3) BDNF*. BDNF is a neurotrophic factor that supports the differentiation, maturation, and survival of neurons [48]. BDNF levels in the serum were measured via ELISA per manufacturer instructions (R&D Systems Inc, Minneapolis, MN, USA).

### Statistical analysis

Values below the level of detection of the assay were assigned half of the corresponding minimum value for each variable. β-amyloid and neurofibrillary tangles were square-root transformed to improve normality. Thereafter, data for IFABP, LBP, and BDNF were standardized such that each variable had a mean of 0 and a standard deviation of one. All statistical analyses controlled for age (age at death for brain pathology, age at sample collection for all the other analyses), sex and education. We first assessed the association of serum IFABP, LBP, and BDNF with risk of death, incident AD dementia and incident MCI using Cox proportional hazards models, controlling for age, sex, and education. Next, using longitudinal cognitive data collected, we applied linear mixed-effects models to test the association of IFABP, LBP, and BDNF with change in global cognition as well as the five cognitive domains (episodic memory, perceptual orientation, perceptual speed, semantic memory, and working memory). In these latter analyses, in addition to controlling for age at baseline, sex, and education, terms for interaction with time were also included. These linear mixed-effects models included subject-specific random terms for the intercept and time. Finally, we assessed the association between IFABP, LBP, and BDNF with continuous measures of the burden of AD pathology, β-amyloid, and neurofibrillary tangles, using linear regression, adjusted for demographics. Subsequently, a logistic regression was used to assess the association of IFABP, LBP, and BDNF with the presence of cortical Lewy bodies.

This was a pilot study; therefore, we performed a series of power analyses to estimate sample sizes needed for a larger study in the future. The power of each of the models of the association of a biomarker with an outcome depends on an effect size (defined below for each of the models), on the number of variables controlled for (i.e., 3), and on the proportion of the variation in the biomarker explained by the three variables controlled for (i.e., 0.05, as guided by the data). The effect size for Cox regression when the outcome is time to the occurrence of an event is the hypothesized hazard ratio (**HR**); the proportion of persons (π_1_) for whom the event of interest is observed (not censored) also influences power. Guided by our data, we calculated the power with the HR = 0.84 and 03C0_1_ = 0.8. The effect size for logistic regression of the binary outcome presence of AD dementia depends on the hypothesized odds ratio (**OR**) per standard deviation (**SD**) of the biomarker. Again, we are guided by the data and considered an OR = 0.7, which corresponds to a probability of AD dementia of 0.4 if the biomarker is 1 SD higher than a reference group in which the probability of AD dementia is 0.5. The effect size for linear regression when the outcome is a continuous measure of the neuropathologies is the percentage of variation in the neuropathology explained by the biomarker (03C0_2_). We calculated the power for an effect size π_2_ = 5%. All tests were 2-sided at a significance level of 0.05.

Power calculations were performed using PASS 2008 [49]. All other statistical analyses were programmed in SAS version 9.4 (SAS Institute, Inc., Cary, NC, USA) and R [50].

### Ethics approval

Clinical and postmortem data and biospecimens used in this study were obtained from the Rush Alzheimer’s Disease Center (**RADC**). This study was reviewed and deemed exempt by the Rush University Medical Center Institutional Review Board. The RADC protocols and collection of data in this study was performed in accordance with the ethical standards of Rush University Medical Center and with the 1964 Helsinki declaration and its later amendments or comparable ethical standards.

## Results

Characteristics of the study participants are given in Table 1. Briefly, of the 88 participants, 30% were male, the mean age was 76.4 years of age at baseline with a mean length of follow up 13.7 years. Of the 88 participants, 65 (74%) developed MCI and 41 (47%) developed AD dementia. Cognitive testing was conducted annually. All 41 participants who were diagnosed with AD were first diagnosed with MCI. Twenty-four participants were diagnosed as MCI, nine with amnestic MCI and 18 with non-amnestic during annual testing (three participants had a combination of amnestic and non-amnestic MCI).

**Fig 1:**
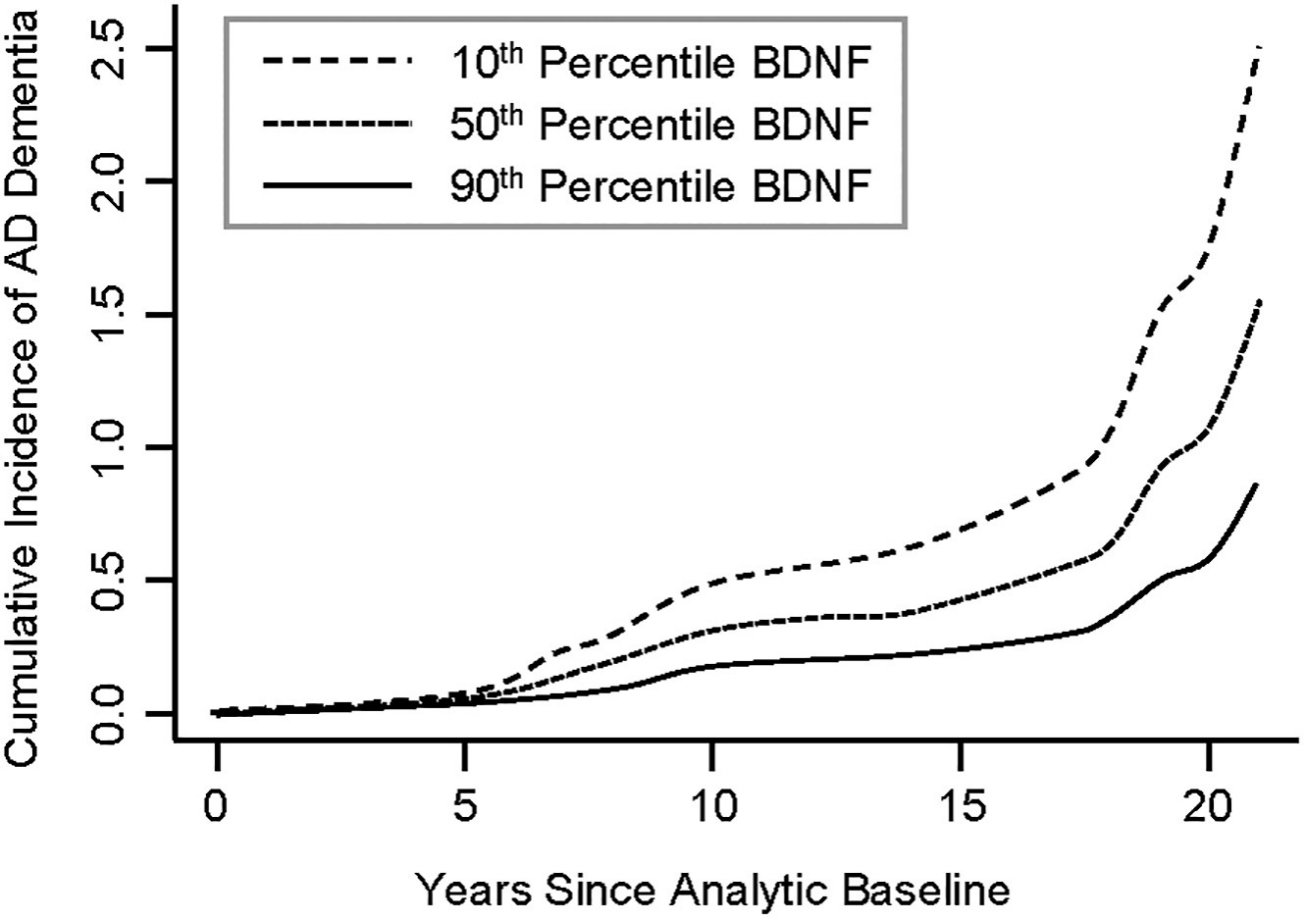
Higher serum BDNF is associated with lower cumulative incidence of AD dementia. Dashed Line: 10^th^ percentile of BDNF, Dotted Line: 50^th^ percentile of BDNF, Solid Line: 90^th^ percentile of BDNF. BDNF was standardized. BDNF, brain derived neurotrophic factor.

**Fig 2:**
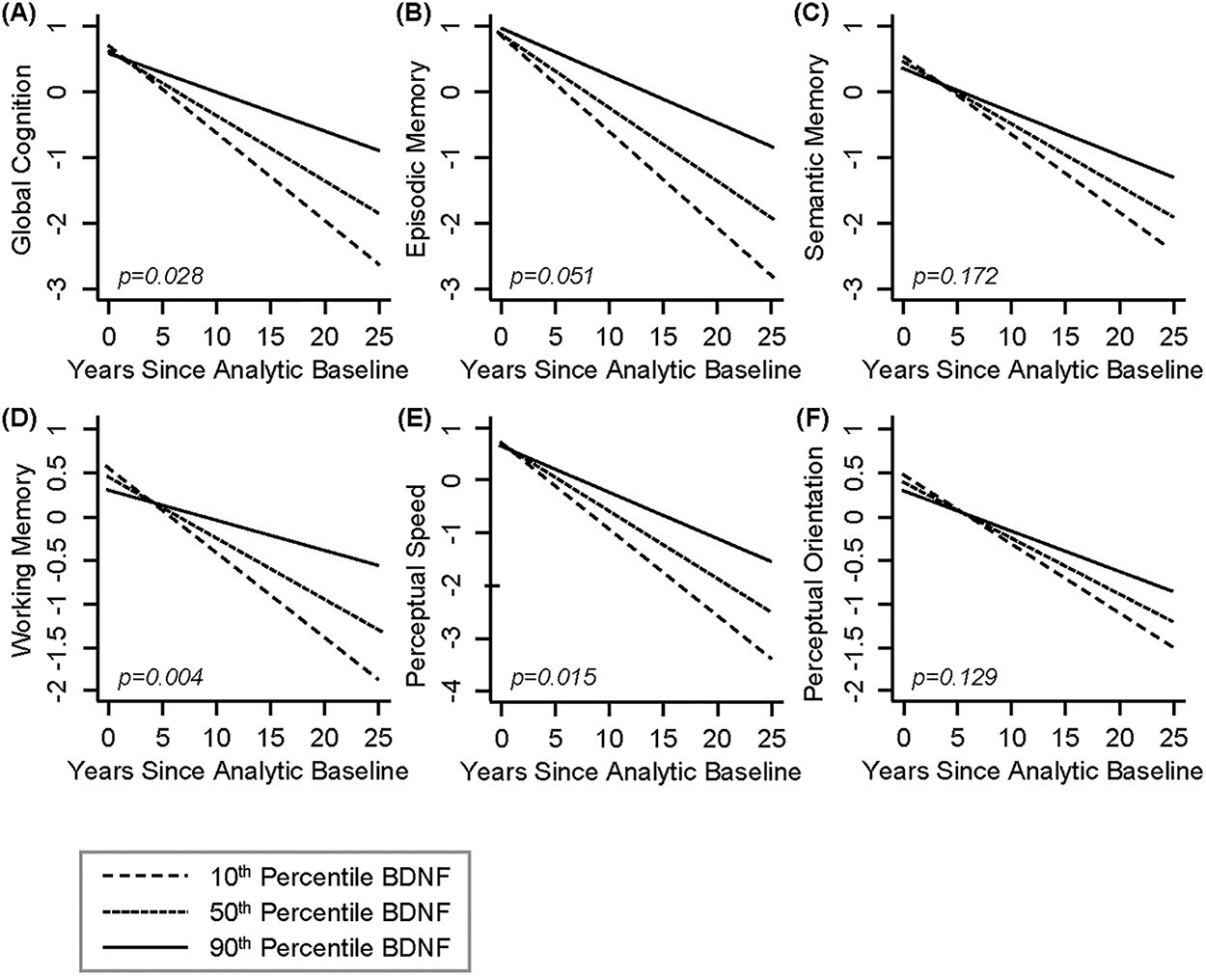
Higher serum BNDF is associated with slower rate of cognitive decline over time. Dashed Line: 10^th^ percentile of BDNF, Dotted Line: 50^th^ percentile of BDNF, Solid Line: 90^th^ percentile of BDNF. **(A)** Global Cognition, **(B)** Episodic Memory, **(C)** Semantic Memory, **(D)** Working Memory, **(E)** Perceptual Speed, and **(F)** Perceptual Orientation. BDNF was standardized. BDNF, brain derived neurotrophic factor.

First, we examined the relationship between BDNF and risk of death, incidence of AD dementia, and MCI. There were no associations between BDNF and risk of death and incident MCI. However, BDNF was associated with incident AD dementia (HR = 0.699, 95% CI: 0.496–0.985, p = 0.041; Table 2). Fig 1 shows the cumulative incidence of AD dementia for participants with 10^th^ percentile, 50^th^ percentile and 90^th^ percentile of BDNF, indicating that participants with a higher level of serum BDNF were less likely to develop AD dementia compared with those who had a lower level of BDNF. We then examined the association between BDNF and change in global cognition, and in secondary analyses, the five cognitive domains. BDNF was associated with change in overall global cognition (interaction p = 0.028), as well as working memory (interaction p = 0.004), and perceptual speed (interaction p = 0.015), but not with episodic memory (interaction p = 0.051), semantic memory (interaction p = 0.172), nor perceptual orientation (interaction p = 0.129) (Table 3). Fig 2 demonstrates that subjects with a higher level of serum BDNF exhibited a slower decline in global cognition (Fig 2A), working memory (Fig 2D), and perceptual speed (Fig 2E), compared with those who had a lower level of BDNF. Finally, we examined the association between BDNF and neuropathologies and found no significant associations of BDNF with any of the neuropathologies examined (Table 4). The power in this study was limited. Autopsy and neuropathologic assessments from 67 subjects were used in this analysis; however, this sample size yields less than 50% statistical power to detect 5% of variation. Power calculation suggests that a sample size of 144 is needed to test these associations with 80% power.

**Table 2:** Association with risk of incident MCI, AD and death.

		HR (95% CI)	P-value
IFABP	Death	1.103 (0.885–1.373)	0.383
	MCI	0.871 (0.671–1.131)	0.301
	AD	0.898 (0.633–1.274)	0.547
LBP	Death	0.871 (0.661–1.147)	0.324
	MCI	1.093 (0.821–1.455)	0.541
	AD	0.965 (0.677–1.374)	0.841
BDNF	Death	0.828 (0.634–1.082)	0.167
	MCI	0.810 (0.613–1.071)	0.140
	AD	0.699 (0.496–0.985)	0.041*

In all analyses, a Cox regression was used, controlling for age at study baseline, sex, and education. IFABP, LBP, and BDNF were standardized in all the analyses. AD, Alzheimer’s disease; BDNF, brain derived neurotrophic factor; CI, confidence interval; HR, hazard ratio; IFABP, intestinal fatty acid binding protein; LBP, lipopolysaccharide binding protein; MCI, mild cognitive impairment.

**Table 3:** Association of BDNF, IFABP, LBP with cognition (global cognition and domains: Episodic memory, semantic memory, working memory, perceptual speed, perceptual orientation).

		Level		Slope	
		Estimate (SE)	P-value	Estimate (SE)	P-value
IFABP	Global cognition	0.031 (0.057)	0.590	0.014 (0.012)	0.221
	Episodic memory	0.025 (0.057)	0.662	0.021 (0.014)	0.134
	Semantic memory	-0.040 (0.070)	0.571	0.014 (0.013)	0.275
	Working memory	0.001 (0.085)	0.993	0.007 (0.008)	0.376
	Perceptual speed	0.087 (0.083)	0.302	-0.001 (0.012)	0.951
	Perceptual orientation	0.049 (0.073)	0.504	0.002 (0.008)	0.791
LBP	Global cognition	-0.046 (0.063)	0.466	0.004 (0.013)	0.760
	Episodic memory	-0.024 (0.063)	0.701	-0.001 (0.016)	0.943
	Semantic memory	-0.044 (0.076)	0.561	0.020 (0.015)	0.174
	Working memory	-0.112 (0.092)	0.227	0.012 (0.009)	0.192
	Perceptual speed	0.018 (0.090)	0.841	0.002 (0.013)	0.879
	Perceptual orientation	-0.075 (0.078)	0.345	0.005 (0.009)	0.567
BDNF	Global cognition	-0.036 (0.059)	0.539	0.026 (0.012)	0.028*
	Episodic memory	0.018 (0.059)	0.760	0.028 (0.014)	0.051
	Semantic memory	-0.057 (0.071)	0.423	0.018 (0.013)	0.172
	Working memory	-0.099 (0.086)	0.254	0.023 (0.008)	0.004**
	Perceptual speed	-0.024 (0.083)	0.770	0.027 (0.011)	0.015*
	Perceptual orientation	-0.057 (0.072)	0.433	0.012 (0.008)	0.129

Analyses were performed using linear mixed-effects models, adjusting for age at baseline, sex and education. IFABP, LBP, and BDNF were standardized in all the analyses. Slope represents change per year. BDNF, brain derived neurotrophic factor; IFABP, intestinal fatty acid binding protein; LBP, lipopolysaccharide binding protein; SE, standard error.

*P-value < 0.05,

**P-value < 0.01 in bold means statistical significance.

**Table 4:** Association of BDNF, IFABP, and LBP with neuropathologies (global AD pathology, β-Amyloid, Tau Tangles, Lewy bodies).

		Estimate (SE)	P-value
IFABP	Global AD Pathology	0.009 (0.081)	0.913
	β-Amyloid	-0.101 (0.146)	0.490
	Tau Tangles	0.093 (0.177)	0.602
	Lewy Bodiesⱡ	-0.292 (0.331)	0.379
LBP	Global AD Pathology	-0.031 (0.078)	0.695
	β-Amyloid	-0.168 (0.141)	0.238
	Tau Tangles	0.018 (0.172)	0.916
	Lewy Bodiesⱡ	0.126 (0.299)	0.673
BDNF	Global AD Pathology	-0.024 (0.078)	0.754
	β-Amyloid	-0.072 (0.142)	0.611
	Tau Tangles	-0.052 (0.172)	0.764
	Lewy Bodiesⱡ	0.109 (0.297)	0.713

All statistical analyses, were controlled for age at death, sex, and education. IFABP LBP, and BDNF were standardized in all analyses. Linear regression was used unless otherwise stated.

^ⱡ^Logistic regression. AD, Alzheimer’s disease; BDNF, brain derived neurotrophic factor; IFABP, intestinal fatty acid binding protein; LBP, lipopolysaccharide binding protein; SE, standard error.

Next, we examined the association between IFABP and LBP with risk of death, incident AD dementia, MCI, and changes in global cognition including the five domains. There were no associations between markers of barrier integrity and risk of death, incident AD dementia, or incident MCI (Table 2). Likewise, there were no associations between IFABP and LBP with change in global cognition, and in secondary analyses, change in any of the five cognitive domains (Table 3). We then examined the association of IFABP and LBP with neuropathologies including global AD pathology, β-amyloid, tau tangles, and Lewy Bodies (Table 4) and found no associations. Note that the power for these analyses was limited. With a sample size of 88, the statistical power was less than 35% to detect a hazard ratio of 0.84, and less than 40% to detect an odds ratio of 0.7. Power calculation suggests that a sample size of 319 is needed for Cox analysis and 261 for logistic analysis to test these associations with 80% power.

## Discussion

Our data demonstrate that serum BDNF is associated with cognitive decline and the development of AD dementia. Specifically, BDNF is lower in individuals who eventually develop AD dementia and BDNF is associated with the rate of change in global cognition and most strongly associated with the declining cognitive abilities of working memory and perceptual speed. These findings are in keeping with the literature. Lower levels of BDNF in both the brain and serum (mRNA, protein) are associated with AD [25, 28–30, 38, 51, 52]. Samples analyzed in this study were collected when participants were cognitively normal, before the development of impairment suggesting that a reduction in BDNF may occur very early in the pathogenesis of neurodegeneration. Indeed, BDNF is decreased in the pre-clinical stages of AD and serum BDNF levels are associated with cognition and may predict a slower rate of cognitive decline [36, 53]. To date, a single study has shown that dementia-free individuals with higher BDNF are less likely to develop dementia and AD [39]. Our findings recapitulate the outcomes of this previous study and demonstrate that serum BDNF, assessed when subjects were cognitively normal, is associated with cognitive decline during 10 years after sample collection. These findings may have important public health consequences and suggest that BDNF may be a robust clinical biomarker to identify older adults at risk for developing AD dementia which can facilitate early interventions to prevent dementia.

Evidence indicates that inflammation is a critical factor that precedes the development of AD [54]. Thus, we proposed that intestinal barrier dysfunction (which leads to systemic and neuroinflammation) might precede incident AD or MCI, cognitive dysfunction, and neuropathology. However, we did not find any significant associations between markers of intestinal barrier integrity (IFABP, LBP) with risk of death, incident AD or MCI, cognitive dysfunction, or neuropathology. It could be that intestinal barrier dysfunction does not precede these events (and is not a causative factor) or it could be interpreted that the markers that we selected to assess barrier integrity were not appropriate to capture the specific type of barrier dysfunction that is present prior to development of incident AD dementia, MCI, or cognitive dysfunction. In this study, we assessed IFABP (which indicates intestinal epithelial cell damage) and LBP (which indicates immune response to intestinal barrier dysfunction); however, a disrupted apical junctional complex (**AJC**) is the most common cause of intestinal barrier dysfunction. Unfortunately, there is no reliable serum marker to assess AJC integrity in the intestine [55–57], therefore we cannot assess this potential mechanism. If intestinal barrier dysfunction is due to disrupted AJC, then additional analyses will need to be conducted once these tools are developed and only then can we conclusively determine if intestinal barrier dysfunction precedes incident AD dementia, MCI, cognitive dysfunction, and/or neuropathology. Nonetheless, based on the data generated in this pilot study, loss of intestinal barrier integrity does not precede diagnosis of AD dementia or MCI, cognitive decline, or neuropathology.

There are several limitations in this study. As already mentioned, our assessments of the intestinal barrier were limited to measurements of serum IFABP and LBP [45] which may not accurately represent the type of barrier dysfunction that precedes cognitive decline. The development of additional assays in the future to detect systemic markers of changes in AJC may be useful in determining if another type of barrier dysfunction is present. The other alternative is to examine intestinal or sigmoid biopsy samples for AJC proteins but these samples are not available in the participants that were included in this study. Finally, our sample size was small and the statistical power was limited for some of the analyses we performed. Some of the insignificant findings may be a result of insufficient statistical power (supported by power calculations); therefore, findings need to be validated in a larger cohort. Additionally, the MCI group was heterogenous and included a mix of amnestic, non-amnestic, and a combination of amnestic/non-amnestic participants, and this variability may reduce power. Future studies appropriately powered to evaluate these sub-types are needed to more fully characterize these transitions.

Taken together, this pilot study suggests that a reduction in BDNF may occur early in the pathogenesis of neurodegeneration and may be a critical event leading to AD and/or may be a useful biomarker to identify subjects at risk for AD. Lastly, the data suggest that intestinal barrier integrity does not appear to be associated with the development of AD clinical or pathologic phenotypes. Barrier integrity is suggested to be compromised in AD which means intestinal involvement may occur later in disease pathology. While loss of barrier integrity in AD is potentially not involved in the early events leading to MCI or AD, it may promote neuroinflammation and amyloid-β overproduction leading to AD progression.
